# Contact investigations for antibiotic-resistant bacteria: a mixed-methods study of patients’ comprehension of and compliance with self-sampling requests post-discharge

**DOI:** 10.1186/s13756-023-01277-1

**Published:** 2023-08-10

**Authors:** Anneloes van Veen, Dominique L. A. Lescure, Suzanne J. C. Verhaegh, Inge de Goeij, Vicki Erasmus, Ed F. van Beeck, Aimée Tjon-a-Tsien, José Splinter, Jan C. Christiaanse, Marjolein Damen, Elisabeth G. W. Huijskens, Sunita Paltansing, Michiel van Rijn, Jacobien Veenemans, Margreet C. Vos, Juliëtte A. Severin, the Infection Prevention and Antimicrobial Resistance Care Network South-western Netherlands

**Affiliations:** 1 Department of Medical Microbiology and Infectious Diseases, Erasmus MC University Medical Center, P.O. Box 2040, 3000 CA Rotterdam, The Netherlands; 2 Department of Public Health, Erasmus MC University Medical Center Rotterdam, P.O. Box 2040, 3000 CA Rotterdam, The Netherlands; 3 Department of Infectious Disease Control, Municipal Public Health Service Rotterdam-Rijnmond, Rotterdam, The Netherlands; 4 Department of Medical Microbiology and Infection Prevention, Franciscus Gasthuis and Vlietland, Rotterdam, The Netherlands; 5 General Practitioners Practice ‘Nieuwe Maas’, Schiedam, The Netherlands; 6 Department of Medical Microbiology, Maasstad General Hospital, Rotterdam, The Netherlands; 7 Department of Medical Microbiology, Albert Schweitzer Hospital, Dordrecht, The Netherlands; 8 Department of Medical Microbiology and Infectious Diseases, Ikazia Hospital, Rotterdam, The Netherlands; 9 Department of Infection Prevention, Admiraal de Ruyter Hospital, Goes, The Netherlands

**Keywords:** Antimicrobial drug resistance, Compliance, Comprehension, Contact tracing, Health communication, Health literacy, Self-examination

## Abstract

**Background:**

Contact investigation is an important tool to identify unrecognized patients who are colonized with antibiotic-resistant bacteria. Many Dutch hospitals include already discharged contact patients by sending them a self-sampling request at home, incl. an information letter and sampling materials. Each hospital composes these information letters on their own initiative, however, whether discharged patients comprehend and comply with these requests remains unclear. Therefore, the aim was to provide insight into patients’ comprehension of and self-reported compliance with self-sampling requests post-discharge.

**Methods:**

This mixed-methods study was performed in eight Dutch hospitals. First, the Common European Framework of Reference (CEFR) language level of self-sampling request letters was established. Second, a questionnaire about patients’ comprehension of the letter, self-reported compliance, and reasons for compliance or non-compliance were sent to patients that received such a request in 2018/2019. Finally, a random selection of questionnaire respondents was interviewed between January and March 2020 to gain additional insights.

**Results:**

CEFR levels of 15 letters were established. Four letters were assigned level B1, four letters B1–B2, and seven letters B2. The majority of patients reported good comprehension of the letter they had received. Conversely, some respondents indicated that information about the bacterium (18.4%), the way in which results would be communicated (18.1%), and the self-sampling instructions (9.7%) were (partially) unclear. Furthermore, self-reported compliance was high (88.8%). Reasons to comply were personal health (84.3%), the health of others (71.9%), and general patient safety (96.1%). Compliant patients appeared to have a need for confirmation, wanted to protect family and/or friends, and felt they were providing the hospital the ability to control the transmission of antibiotic-resistant bacteria. Although a limited number of non-compliant patients responded to the questionnaire, it seemed that more patients did not comply with self-sampling requests when they received a letter in a higher CEFR-level (B2) compared to a lower CEFR-level (< B2) (9.8% vs. 2.5%, *P* = 0.049).

**Conclusions:**

This study showed an overall good comprehension of and high self-reported compliance with self-sampling requests post-discharge. Providing balanced information in self-sampling request letters has the potential to reduce patient’s ambiguity and concerns, and can cause increased compliance with self-sampling requests.

**Supplementary Information:**

The online version contains supplementary material available at 10.1186/s13756-023-01277-1.

## Background

Antimicrobial resistance (AMR) poses a significant and growing threat to global public health. An important infection prevention and control (IPC) measure to prevent or reduce the transmission of antibiotic-resistant bacteria, such as methicillin-resistant *Staphylococcus aureus* (MRSA) or highly-resistant microorganisms (HRMO), is contact investigation. In healthcare facilities, this procedure is started (either in endemic or epidemic settings) after the unexpected detection of an MRSA or HRMO in a clinical culture of a patient for whom isolation precautions were not applied [1]. Other patients who have been in close contact with the positive index patient, e.g., by staying in the same room or on the same ward, are then screened for carriage of the specific antibiotic-resistant bacterium [1].

To this day, international guidelines for a variety of antibiotic-resistant bacteria are inconclusive about whether and when screening cultures should be taken to identify carriers [2, 3]. The European Society of Clinical Microbiology and Infectious Diseases (ESCMID) guideline, for example, does not provide recommendations on contact investigations in case of an unexpected multidrug-resistant Gram-negative bacteria (MDR-GNB) positive patient neither on definitions of patients or patient groups to be included in a contact investigation [2, 3]. On the other hand, the Swiss guideline on vancomycin-resistant enterococci [4] and the Dutch guidelines on HRMO [1] and MRSA [5] offer guidance with regard to when to perform and whom to include in contact investigations for these microorganisms. Recommendations on whom to include are not necessarily limited to patients that are still admitted in the hospital when a positive index patient is identified, but rather all patients who have been in close proximity to the index patient during their admission [1, 4]. This definition, therefore, also comprises patients who have since the unexpected detection been discharged from the hospital. In the Netherlands, a country with an overall low prevalence of antibiotic-resistant bacteria, the inclusion of discharged patients in MRSA and HRMO contact investigations has, therefore, become part of routine patient care and outbreak management [6–8].

There are different ways in which discharged patients can be screened, such as through home screenings by visiting healthcare workers or by screening in an outpatient clinic [6, 9, 10]. However, most Dutch hospitals invite discharged patients by sending a letter with information, instructions and materials for self-sampling to patients’ homes, after which the swabs can be returned to the hospital by regular mail. Dutch hospitals, therefore, rely on written communication in order to empower patients to make an informed choice, while simultaneously encouraging patients to participate in these contact investigations.

When written health information is difficult to comprehend by persons with low/limited health literacy, this can contribute to lower levels of protective behaviours, such as health screening participation [11–15]. Similarly, a lack of knowledge and understanding of the importance of screening for carbapenemase-producing Enterobacterales (CPE) negatively influenced the acceptability of such screening [16]. Previous research found good acceptance of screening for CPE during hospitalization, either performed by nurses or through self-sampling, and screening of discharged contact patients for vancomycin-resistant enterococci through home screenings by visiting nurses [10, 16]. However, what the effect was of the approach followed within the South-western region of the Netherlands, in which each hospital composed self-sampling request letters and instructions on their own initiative in order to invite patients to participate in MRSA and HRMO contact investigations, in terms of patients’ comprehension of and compliance with such self-sampling requests has so far never been evaluated.

Therefore, the aim of this study was (1) to explore the language level and content of hospitals’ self-sampling request letters, (2) to provide insight into patients’ comprehension of these self-sampling request letters, (3) to investigate their self-reported compliance with self-sampling requests post-discharge, (4) and to provide insight into patients’ attitudes towards and reasons behind compliance or non-compliance.

## Methods

### Setting

The South-western region of the Netherlands consists of eleven hospitals, all of which are affiliated with the Infection Prevention and Antimicrobial Resistance Care Network Southwest Netherlands (‘IP & ABR Zorgnetwerk Zuidwest-Nederland’). Ten out of eleven hospitals participated in this study. The non-participating hospital is a hospital specialized in ophthalmology in which contact investigations are unusual. The IP & AMR care network was established in 2015 as part of the Dutch AMR National Action Plan [17]. The South-western region has approximately two million inhabitants, is characterized by a multicultural population and has a relatively high number of (health) illiterate individuals (ranging between 13 and 16%) [18]. All hospitals within this region are acute care hospitals, which include one university hospital, five teaching hospitals and four non-teaching hospitals (approx. all together 4800 beds, range 219–1125 beds).

### Self-sampling request letters

The hospitals were asked to provide all their self-sampling request letters for MRSA and HRMO, which were sent to discharged contact patients in 2018/2019. Subsequently, each self-sampling request letter was assessed by one of three external communication experts whom established the Common European Framework of Reference (CEFR) language levels and analyzed the content of these self-sampling request letters following a predefined framework. The CEFR language levels are accepted as an international standard for grading language users’ proficiency, whereby six levels are grouped into three categories (from lower to higher level): basic (A1–A2), independent (B1–B2), and proficient users (C1–C2) [19].

### Questionnaire development and composition

The research team developed the questionnaire during multiple discussion rounds. A validated questionnaire was not found in scientific literature, but the questionnaire was inspired by Currie et al. [16] and Merchant et al. [20], and initially included more than 40 questions. The draft was subsequently simplified (written in A2 CEFR-level) and shortened by two health literacy experts to improve questionnaire comprehension for patients with limited health literacy, thereby allowing for maximum questionnaire response. The simplified questionnaire was tested for comprehensibility by a selected group of health illiterate individuals who did not have any prior knowledge regarding antibiotic-resistant bacteria and contact investigations. This test round led to minor alterations in the questionnaire. The final questionnaire contained 23 multiple choice questions related to patient characteristics, patients’ comprehension of the self-sampling request letter they had received in 2018/2019, patients’ self-reported compliance or non-compliance with the self-sampling request (due to unavailability of regional self-sampling compliance rates, self-reported compliance was asked), and their reasons for compliance or non-compliance. The standard informed consent form was also adapted to CEFR-level A2.

### Semi-structured interviews

Respondents who were willing to provide additional clarification regarding their answers during an interview, could provide their consent by writing their contact details at the end of the questionnaire. Only respondents who gave their consent were eligible for a follow-up interview. A concise semi-structured interview was performed with a selection of those respondents to gain in-depth insights. The interview guide (available in Additional file 1) was developed by three researchers (AV, VE, JS). Most interview questions were originally intended for the questionnaire, but were moved to the interview due to the length of the questionnaire. The interview guide consisted of 11 questions that focused mainly on patients’ perceptions of the self-sampling request letter and patients’ reasons for compliance or non-compliance with a self-sampling request.

### Data collection

Hospitals were eligible to participate when self-sampling request letters were sent to discharged contact patients for MRSA and HRMO contact investigations in 2018/2019. The infection prevention teams of the ten hospitals were asked to make a random selection of 100 patients to whom they had sent a self-sampling request letter in 2018/2019. Furthermore, the sample of patients was to be chosen from contact investigations for the antibiotic-resistant bacteria for which most commonly a contact investigation among discharged patients was conducted, including multidrug-resistant *Acinetobacter baumannii* (MDR-AB), CPE, multidrug-resistant *Pseudomonas aeruginosa* (MDR-PA), MRSA and vancomycin-resistant *Enterococcus faecium* (VRE), with preferably 20 patients per antibiotic-resistant bacterium. Patients were eligible to participate if they were aged 18 years or older. Questionnaires with the majority of answers (≥ 70%) related to comprehension and reasons for compliance or non-compliance missing were not eligible for inclusion. Between November 2019 and January 2020, selected patients received a package at their home address, which contained a patient information letter about the study, a paper-based questionnaire (an electronic version was also made available), and the original self-sampling request letter patients had received in the 2 years prior to serve as a memory aid.

The follow-up semi-structured interviews were conducted as unscheduled telephone calls instead of face-to-face interviews. This was the method of choice, because of practicality (i.e., geographical dispersion of hospitals and, therefore, of respondents) and to make follow-up more accessible for the respondents (i.e., less time and effort). Respondents who were willing to participate in the follow-up, were stratified by their previous self-reported compliance or non-compliance. A computerized random selection program was used to select patients from these groups. Respondents were called unannounced and this process was continued until data saturation was reached. All semi-structured telephone interviews took place between January and March 2020 and lasted between 5 and 28 min. One infection prevention specialist and three researchers performed all interviews.

### Data analysis

CEFR language levels and questionnaire data were entered into IBM Statistical Package for the Social Sciences Solutions (SPSS) version 28 (IBM Corp., Armonk, New York, USA), which was used for all quantitative analyses. The actual CEFR-level of the self-sampling request letter was used for each respondent. However, when no self-sampling request letter was assessed for the specific antibiotic-resistant bacterium for which the respondent was requested to self-sample, we used the average CEFR-level of all provided letters by the specific hospital. Inconsistent, incomplete and missing answers from the questionnaires were excluded from the analyses. For descriptive purposes, a median with range, frequencies and percentages were calculated where appropriate. Self-sampling compliance was compared between patients receiving the letter in a higher CEFR-level (B2) to a lower CEFR-level (B1 and B1–B2) using Chi-Square test. A *P* value of < 0.05 was considered statistically significant.

The semi-structured interviews were audio recorded and fully transcribed. Before the start of the interview, the interviewees gave their oral informed consent and were requested to give permission for audio recording. All privacy-related information was removed from the transcripts. Interview transcripts underwent thematic content analysis, using NVivo software, version 10 (QSR International, Doncaster, Australia). To guarantee the reliability and validity of the analyses, two independent researchers (AV and DL) combined phrases to generate categories. All transcripts were analyzed in this manner until no new categories emerged from the data. Subsequently, axial coding was used to look for relationships between open codes and to link categories into overlying themes. Conflicting opinions on categories and themes were discussed and resolved by three researchers (AV, DL and VE).

## Results

Eight hospitals were found eligible for participation in sending out questionnaires. One hospital did not send any self-sampling request letters to discharged patients in 2018/2019. Another hospital did not perform any contact investigations for MRSA or HRMO, but delegated this task to local general practitioners (GPs).

### Self-sampling request letters

One of the eight hospitals did not provide self-sampling request letter(s) for analysis. The CEFR language level of fifteen self-sampling request letters from seven hospitals was established. Four letters (26.7%) were CEFR language level B1, four letters (26.7%) were language level B1-B2 and seven letters (46.7%) were language level B2 (Table 1).

**Table 1:** CEFR language levels^a^ of self-sampling request letters

Hospital	Number of letters	(Average) CEFR-level	Focus of letter(s)	(Median) number of pages	(Median) number of illustrations
1	2	B2	HRMO and MRSA	1	0
2	5	B1	HRMO, MDR *Acinetobacter baumannii,* MDR *Pseudomonas aeruginosa,* MRSA, and VRE	2	0
3	1	B1–B2	HRMO/MRSA	1	0
4	2	B2	HRMO and MRSA	1	0
5	0	–	–	–	–
6	1	B2	MRSA	1	0
7	3	B2	MRSA and VRE	1	0
8	1	B1–B2	MRSA	1	0
Total	15	–	–	–	–

^a^CEFR language levels: basic (A1–A2), independent (B1–B2), and proficient users (C1–C2). *HRMO* highly-resistant microorganism, *MRSA* methicillin-resistant *Staphylococcus aureus*, *MDR* multidrug-resistant, *VRE* vancomycin-resistant *Enterococcus faecium*

### Inclusion

**Fig. 1:**
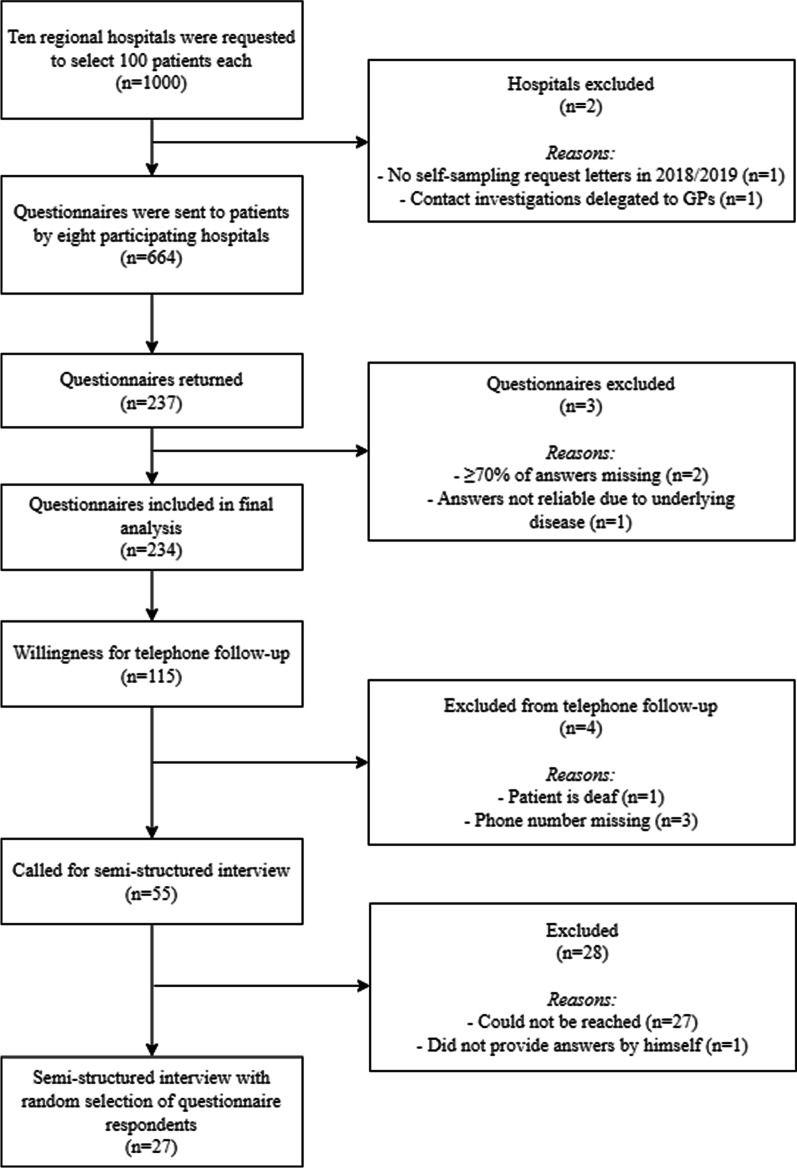
Flowchart of patient inclusion.

Three out of eight hospitals had less than 100 eligible patients for participation. Hence, the questionnaire was sent to a total of 664 patients. The questionnaire response rate was 35.7% (237/664). Two questionnaires were excluded due to missing the majority of answers (≥ 70%), which was one of the inclusion criteria, and one questionnaire was excluded because the patient appeared to have dementia and answers were unreliable. Therefore, 234 questionnaires were eligible for analysis (Fig. 1). Table 2 shows the sample sizes, number of respondents, response rates per hospital and number of respondents per microorganism. The basic characteristics of respondents are detailed in Table 3.

**Table 2:** Sample sizes, number of respondents, response rates and respondents per type of HRMO

Hospital	Sample size	Respondents	Response rates (%)	MRSA	VRE	CPE	MDR-AB	MDR-PA
1	100	51	51.0	18	27	6	–	–
2	100	41	51.0	16	9	–	9	7
3	100	41	41.0	13	26	–	–	2
4	100	32	32.0	10	16	2	4	–
5	100	30	30.0	18	12	–	–	–
6	68	17	25.0	17	–	–	–	–
7	62	22	35.5	5	–	17	–	–
8	34	3	8.8	3	–	–	–	–
Total	664	237	35.7%	100	90	25	13	9

*HRMO* highly-resistant microorganism, *MRSA* methicillin-resistant *Staphylococcus aureus*, *VRE* vancomycin-resistant *Enterococcus faecium*, *CPE* carbapenemase-producing Enterobacterales, *MDR-AB* multidrug-resistant *Acinetobacter baumannii*, *MDR-PA* multidrug-resistant *Pseudomonas aeruginosa*

**Table 3:** Basic characteristics of questionnaire respondents (n = 234)

Respondents' characteristics	N	%
Median age (range) (n = 218)	68 (20–90)	
Gender (n = 234)		
Male	111	47.4
Female	122	52.1
Other	1	0.4
Living conditions (n = 231)		
Independently	224	97.0
In a healthcare facility	7	3.0
Children < 18 years living in household (n = 223)		
Yes	23	10.3
No	200	89.7
Highest educational level (n = 231)		
No education	6	2.6
Primary school	17	7.4
High school	96	41.6
University of applied sciences	96	41.6
University	16	6.9
(Previous) Healthcare worker (n = 172)ª		
Yes	32	18.6
No	140	81.4

ªDue to the questionnaire layout, this question often went unnoticed

One hundred and fifteen respondents were willing to participate in telephone follow-up. Data saturation was reached after conducting interviews with 27 respondents, of whom several corresponding quotes are shown in Table 4.

**Table 4:** Illustrative comments from thematic content analysis of semistructured interviews

Topic	Illustrative comment
Content letter: positive opinion	But the letter itself was clear and the instructions on how to take the swabs was also clear. […] And the material was also clear. A precise instruction on what to do, the instruction material was fine.
Content letter: negative opinion	
HRMO information	[…] symptoms of the bacterium are not clear […]
Self-sampling instructions	Slightly more specific explanation. For someone who never does this [taking swabs] it remains difficult to independently carry this out
Receiving results	The letter states: in the event of a positive result you will be informed by telephone. But for a negative result, the information is missing
Timing	But yes, so, yes, I assume that those letters are sent fairly soon after it becomes known that, eh, one of your roommates did indeed carry that bacterium
	[…] uh, the point in time was very far away. […]. Weeks! Because I already thought: well, if I really carry it, then the rest of my environment is also screwed
Reasons for patients to comply with the self-sampling request	
Personal	Well I really wanted to know if I was carrying that MRSA bacteria
Others	Well, no, I, I thought it was normal to participate. I mean, eh, if I can help someone else with it. That was more my choice
General	Well, well, if such a bacteria is…is found. And if they can't get a grip on it somehow, that is important, yes

### Comprehension of the self-sampling request letter

The majority of questionnaire respondents reported good comprehension of the self-sampling request letter they had received (Table 5). While some patients did not express a strong opinion regarding the content of the received letter or did not have an opinion at all, others clearly stated what they liked or disliked during interviews. On the positive side, respondents indicated that the letter was understandable, well readable and clear to them. For example, the letter provided clear and precise instructions on how to self-sample. Some patients also expressed a feeling of reassurance, because, based on the information in the letter, they believed that the consequences of colonization with an antibiotic-resistant bacterium would not be that severe.

**Table 5:** Patients' comprehension of the self-sampling request letter (n = 234)

Question	N	%
Did you have time to read this letter? (n = 230)		
Yes	225	97.8
No	5	2.2
Was the screening for the detection of the bacterium clear at the time? (n = 228)		
Yes	205	89.9
No	8	3.5
A little	15	6.6
Did you find the information about the bacterium clear at the time? (n = 229)		
Yes	187	81.7
No	13	5.7
A little	29	12.7
Did you find the information about taking the swabs clear at the time? (n = 227)		
Yes	205	90.3
No	7	3.1
A little	15	6.6
Was it clear how you would get the results? (n = 227)		
Yes	186	81.9
No	20	8.8
A little	21	9.3
Would you have liked more information? (n = 220)		
Yes	47	21.4
No	173	78.6
Did you search for more information? (n = 231)		
Yes	56	24.2
No	175	75.8
If so, where? (n = 56)^a^		
Internet	46	
General practitioner	8	
Family/friends	6	
Somewhere else	7	
Have you done things differently to not infect anyone? (n = 228)		
Yes	38	16.7
No	190	83.3
Would you have liked to receive the letter in another language? (n = 230)		
Yes	2	0.9
No	228	99.1

^a^Patients could give multiple answers

On the contrary, questionnaire respondents stated a variety of points they disliked in the received letter. Some patients (18.4%) found the information about the antibiotic-resistant bacterium partially or completely unclear. Particularly, the risks associated with carriage and the symptoms to be aware of in case of developing an infection caused by the antibiotic-resistant bacterium were unclear to patients. While self-sampling instructions were explicitly regarded as being clear by the majority, other respondents (9.7%) found these instructions partially or completely unclear. Also, respondents could not always distill from the information in the letter what the situation practically meant for them personally, e.g., whether scheduled appointments could take place or not until the self-sampling results would become available. Furthermore, about one fifth (18.1%) of respondents found it partially or completely unclear how they would receive either a positive and/or a negative result. In general, some patients (21.4%) would have liked more information and a quarter of the patients (24.2%) actually searched for more information, which was done mostly online.

Another theme mentioned by respondents was the timing of the letter. In general, patients trusted their hospital in being accurate and as swift as possible in contacting them with the letter. However, not all patients were appreciative of the moment they received the self-sampling request, which was from their perspective quite late after discharge from the hospital.

Interestingly, although the self-sampling request letter was mostly regarded as well understood, it seemed that the majority of respondents with the lowest levels of education, received the letter in the highest observed CEFR-level B2 (5 out of 6 respondents with no education, 83.3%; 7 out of 14 respondents with primary school, 50.0%; and 45 out of 83 respondents with high school, 54.2%, as their highest educational level). Patients’ comprehension of the self-sampling request letter by CEFR-level of the received self-sampling request letter and educational level is available in Additional file 2.

### Self-reported compliance

Self-reported compliance with self-sampling requests post-discharge was high (206 patients, 88.8%; Table 6). Reasons for patients to comply with the self-sampling request were: personal health (194 patients, 84.3%), the health of other patients (164 patients, 71.9%), and general patient safety (222 patients, 96.1%). With regard to respondents’ own health, the most frequently mentioned underlying reason during interviews was the need for confirmation; the respondent wanted to know whether he or she was carrying the antibiotic-resistant bacterium. Especially when a patient deemed himself/herself in a vulnerable health state, the patient decided to participate because of the belief it could aid in avoiding potential risks associated with the antibiotic-resistant bacterium. Concerning the health of others, respondents complied with their hospital’s request in order to protect e.g., other patients, their relatives and other individuals in general. General patient safety as a reason to participate, was mainly based on the idea of providing hospitals the chance to control transmission to other (hospitalized) patients. Furthermore, the mere request by the hospital to self-sample for MRSA or HRMO detection was in some cases simply sufficient reason to participate. When asked if the obligation to report carriers of certain HRMO to national authorities would affect patients’ compliance, patients indicated that their compliance would not be affected. However, patients did want to be informed on sharing their personal data with national authorities. Of note, in addition to the desired behavior that the letter is aimed at, namely self-sampling compliance, 16.7% of questionnaire respondents also reported a change in their behavior not advocated by the letter. Directly after reading the information in the self-sampling request letter, these respondents would e.g., start washing their hands more often and/or avoid contact with relatives in order to prevent transmission.

**Table 6:** Patients’ self-reported compliance with self-sampling requests and reasons for compliance or non-compliance (n = 234)

Question	N	%
Did you participate in the screening for the bacterium? (n = 232)		
Yes	206	88.8
No	15	6.5
I don't know anymore	11	4.7
If 'yes': who took the swabs? (n = 203)		
I took the swabs myself	160	78.8
I had the swabs taken by someone else	43	21.2
If 'No': why didn't you participate? (n = 13)^a^		
I didn't understand the letter	1	
I didn't think it was necessary	3	
I didn't want to	1	
I find it scary	0	
Something else	9	
Did you think you had the bacterium when you got the letter? (n = 229)		
Yes	18	7.9
No	152	66.4
I don't know	59	25.8
When you received the letter about the contact investigation, did you think "the hospital must have been dirty then"? (n = 231)		
Yes	16	6.9
No	196	84.8
I don’t know	19	8.2
Did you feel sicker after taking swabs? (n = 230)		
Yes	14	6.1
No	202	87.8
I don't know	14	6.1
Did you participate in the screening for detection of the bacterium for your own health? (n = 230)		
Yes	194	84.3
No	27	11.7
I don't know	9	3.9
Did you participate in the screening for detection of the bacterium for the health of other patients in the hospital? For example, not to infect other patients (n = 228)		
Yes	164	71.9
No	51	22.4
I don't know	13	5.7
Do you think screening is important for patient safety? (n = 231)		
Yes	222	96.1
No	1	0.4
I don't know	8	3.5

^a^One patient provided two reasons

On the other hand, patients indicating that they did not comply with the self-sampling request (6.5%) pointed out a variety of reasons for doing so, e.g., not understanding the letter, thinking it was not necessary to participate, or not wanting to participate (Table 5). Although the number of non-compliant patients which responded to the questionnaire was limited, it appeared that they were lower educated compared to patients declaring compliance with self-sampling requests (Additional file 2: Table S1). Furthermore, they found the information from the self-sampling letter more unclear compared to their counterparts in four areas: the information about the screening for the detection of the bacterium (35.7% of non-participants vs. 7.8% of participants), the information about the bacterium (28.6% of non-participants vs. 16.7% of participants), the information about taking the swabs (38.5% of non-participants vs. 5.9% of participants) and the information about receiving the results (50.0% of non-participants vs. 14.9% of participants). Also, it appeared that more patients did not comply with self-sampling requests when they received a letter in a higher CEFR-level (B2) compared to a lower CEFR-level (< B2) (9.8% vs. 2.5%, *P* = 0.049).

## Discussion

This is the first study to investigate the practice of requesting discharged patients to self-sample in the context of contact investigations for MRSA and HRMO, as performed by the hospitals in our IP & AMR Care Network. Our findings show an overall good comprehension of the self-sampling request letter patients had received and a self-reported compliance of 88.8%. A Swiss study [10] reported similar findings when screening VRE contact patients post-discharge. Overall, they found good patient acceptance and a screening compliance of 87.1%, however, screening was performed by nurses who visited patients’ homes instead of by requesting patients to self-sample, and the Swiss study reported the compliance rate as opposed to the self-reported compliance rate in our study [10].

Reasons to comply with the self-sampling requests post-discharge were related to patients’ personal health, the health of other patients, family and/or friends, and patient safety in general. Besides positive findings, our research also identified several key areas of ambiguity in the provided health information: information related to possible symptoms and risks associated with carriage of an antibiotic-resistant bacterium and information on how results would be received was partially or completely unclear. This information was especially reported to be more unclear by declared non-compliant individuals.

A possible explanation for this observation can be derived from the Protection Motivation Theory [21]. According to this theory, a person’s behavior is predicted by their intention to perform that behavior. The perceived severity of a health threat and the perceived probability of its occurrence (i.e., vulnerability) influence the intention to perform protective behaviors, e.g., perfoming self-sampling post-discharge in the context of an MRSA or HRMO contact investigation [21, 22]. Also, confidence in performing the required behavior (i.e., self-efficacy) and the belief that this behavior will be effective in reducing or eliminating a health threat (i.e., response efficacy) enhance the intention to perform protective behaviors. Components of this theory (vulnerability, self-efficacy, and response efficacy) were found to be predictors of protective behaviors, including (self-)testing, for other infectious diseases, such as *Chlamydia trachomatis* (CT) and COVID-19 [23, 24]. With regard to requesting discharged contact patients to participate in contact investigations for antibiotic-resistant bacteria, this theory highlights the importance of sending out carefully composed self-sampling request letters. When the severity, vulnerability and/or response efficacy are perceived as unclear while patients read the self-sampling request letter, these patients might be less inclined to self-sample. Therefore, according to this theory, it seems important for all recipients of such requests, and especially for (health) illiterate individuals, to e.g., adjust the language level of provided health information and/or to accompany self-sampling instructions with illustrations.

In the context of (increasing) inter-hospital patient transfers, during which patients may bring antibiotic-resistant bacteria with them, it is also important to develop uniformity of information provision, screening policies and IPC measures across a certain geographic area [25, 26]. For example, within our IP & AMR Care Network it became clear that patients were having difficulties with understanding the variety of IPC measures that were imposed on them and with differences in information for the same antibiotic-resistant bacterium when transferred to another hospital. Therefore, the authors believe that a regional or national approach can be of added value for bringing a clear and consistent message across when inviting discharged contact patients to self-sample.

A strength of this study is the multicenter design. This provided us with the opportunity to approach a large number of patients from a variety of contact investigations for which different self-sampling request letters were used. Second, our sequential mixed-methods approach allowed us to explore and validate quantitative questionnaire findings with qualitative data from semi-structured interviews and provided us with in-depth information on patients’ considerations for compliance or non-compliance and on patients’ needs with regard to self-sampling request letters. We translated these findings into a toolbox with recommendations for drafting such letters (Box 1).

This study, however, is also subject to several limitations. First, questionnaire respondents seem to not reflect the regional population structure, i.e., a multicultural population and a relatively high number of (health) illiterate individuals, ranging from 13 to 16% across the South-western region [18]. In 2020, the percentage of highly-educated individuals (highest educational level of university of applied sciences or university) was 31% in the Netherlands and 26% in the South-western region [27, 28], whereas among questionnaire respondents the percentage of highly-educated individuals was considerably higher with 48.5%. These numbers suggest an underrepresentation of low-educated, and possibly (health) illiterate, individuals among questionnaire respondents. With limited/low health literacy known to contribute to higher rates of non-compliance in health screenings [13, 15], the authors believe that the proportion of patients who did not comply with self-sampling requests might be higher among questionnaire non-responders. We tried to reduce the effect of this potential non-response bias by tailoring all research information to suit the linguistic needs of (health) illiterate individuals, however, other measures may be necessary to target this specific group in future research as well as in MRSA or HRMO contact investigations. Due to the underrepresentation of low-educated individuals, the percentages of comprehension and self-reported compliance are likely overestimates, which supports the need to take into consideration the recommendations addressed in Box 1.

**Box 1:** Tips for drafting self-sampling request letters

Involving patients in infection prevention and control starts with proper communication. When requesting contact patients to self-sample for detection of antibiotic-resistant bacteria, informing and reassuring patients should receive high priority. Several points should therefore be taken into consideration when composing a self-sampling request letter:	
1. Information regarding the potential symptoms, risks and consequences associated with the antibiotic-resistant bacteria should be explicitly and clearly stated	
2. The importance of screening (for oneself and others) should become clear from the provided information, e.g., to stress the importance of prevention of transmission to vulnerable patients	
3. Clear and practical step-by-step instructions should be provided so that patients can single-handedly take the necessary swabs at home. Illustrations can help clarify the different steps	
4. Patients should be able to extract what the do’s and dont’s are with regard to hygiene practices until the self-sampling results are known	
5. Self-sampling request letters should inform patients on how the self-sampling results will be received, e.g., by letter or phone. Make sure to always inform patients of the self-sampling results, both in case of confirmed carriage and no carriage	
6. The abovementioned information should be written in a simple, short and concise manner, e.g., one A4-document. A CEFR-level below B2 should, preferably, be used	
7. Inform patients that the letter was sent to them as soon as the unexpected detection was done in order to minimize misunderstanding and frustration with the timing of the letter	
More information can be found at the website of the IP & AMR Care Network South-western Netherlands (in Dutch) [29]	

Second, this study may be subject to desirability bias. Both questionnaire respondents and interviewees may have been inclined to provide socially desirable answers. However, questions were formulated as neutral as possible and anonimity in analyses was ensured to minimize the occurrence of such bias.

Third, self-sampling compliance rates for MRSA and HRMO contact investigations were not available for the participating hospitals in the South-western region of the Netherlands. Therefore, the self-reported compliance was used as a proxy variable instead.

Lastly, findings and implications might not be generalizable to other countries as the way in which patients conceive the provided health information and their motives to participate in these contact investigations might be cultural dependent. Therefore, local and/or national differences should be taken into consideration when requesting patients to participate in contact investigations post-discharge.

Future research validating our results by using a larger sample size of discharged contact patients, including more lower educated individuals, would be of added value. Also, research should be aimed at evaluating the effect(s) of providing more balanced information on discharged patients’ comprehension of and compliance with contact investigations for antibiotic-resistant bacteria on a regional, national and international level. Also, an assessment of the impact thereof on the prevalence of antibiotic-resistant bacteria as well as the cost-effectiveness of including discharged contact patients should be made.

## Conclusions

Contact investigation is an important IPC measure to prevent or reduce the transmission of MRSA and HRMO in healthcare facilities. In the Netherlands, it is common practice to also include discharged patients in contact investigations for these antibiotic-resistant bacteria. Our findings show that requesting these patients to self-sample at home by means of an information letter led to an overall good comprehension of the provided information and high self-reported compliance. An essential component within this procedure is to provide patients with balanced information, which has the potential to reduce patient’s ambiguity and concerns, and can, thereby, cause increased compliance.

### Supplementary Information

**Additional file 1**. **Topic list for semi-structured interviews**. The topic list used during semi-structured interviews with a selection of questionnaire respondents.

**Additional file 2**. **Additional Tables**. Basic characteristics of questionnaire respondents by self-reported compliance. Patients’ comprehension of the self-sampling request letter, asked by four different questions in the questionnaire, by CEFR-level of the received letter and educational level.

## Data Availability

The quantitative dataset and qualitative transcripts generated and analysed during the current study are available from the corresponding author on reasonable request.

## Abbreviations

AMR: Antimicrobial resistance
CEFR: Common European Framework of Reference
CPE: Carbapenemase-producing Enterobacterales
CT: *Chlamydia trachomatis*
ESCMID: European Society of Clinical Microbiology and Infectious Diseases
GPs: General practitioners
HRMO: Highly-resistant microorganisms
IPC: Infection prevention and control
MDR-AB: Multidrug-resistant *Acinetobacter* *baumannii*
MDR-GNB: Multidrug-resistant Gram-negative bacteria
MDR-PA: Multidrug-resistant *Pseudomonas* *aeruginosa*
MRSA: Methicillin-resistant *Staphylococcus* *aureus*
VRE: Vancomycin-resistant *Enterococcus* *faecium*
