# Supplementary material for: Contact investigations for antibiotic-resistant bacteria: a mixed-methods study of patients’ comprehension of and compliance with self-sampling requests post-discharge
Source: Antimicrob Resist Infect Control. 2023 Aug 10;12:77. doi: 10.1186/s13756-023-01277-1 (PMC10413776; doi:10.1186/s13756-023-01277-1)
Supplement: Supplementary file 1 — Additional file 1. Topic list for semi-structured interviews. The topic list used during semi-structured interviews with a selection of questionnaire respondents. [file 13756_2023_1277_MOESM1_ESM.docx]

**Additional file 1: Topic list for semi-structured interviews.**

1. Can you tell us what you liked about this letter?
2. Can you also tell us what you didn’t like about this letter?
3. What was the main reason for you to participate or not to participate in the contact investigation?
4. Perhaps religion or culture can play a role as to whether or not someone takes part in such a screening. May I ask if you have a religion? If so, what religion? Did that play a role in your choice?
5. Can you describe in your own words what a resistant bacterium is?
6. Which resistant bacteria are you familiar with? What was the main source of information that introduced you to these bacteria?
   - Internet
   - Newspaper
   - News
   - Family and/or friends
   - General practitioner
   - Hospital or other healthcare institution
   - Something else
7. Did you think that colonization with resistant bacteria could have consequences for your health? How serious did you think these consequences could be? Do you think differently about that now?
8. Suppose you are a carrier of a resistant bacterium. Would you be concerned about passing it on to another person? If so, who? In your own household or outside your own household?
9. Would you inform others in your private environment? Would you be worried that others will find you ‘dirty’?
10. Would you be concerned that you would receive less quality patient care?
11. Since July 1st, 2019 it is mandatory for hospitals to report carriers of certain special bacteria to the Municipal Health Services (‘GGD’) and National Institute for Public Health and the Environment (‘RIVM’). In case the test confirms that you are a carrier, your culture data, name and address are passed on to these authorities, just as is now the case for tuberculosis for example. What do you think about this? Do you think it is important to know this as a patient?
12. Would this reporting obligation mean that you do or no not participate in a contact investigation request from the hospital? Why or why not?
