# Supplementary material for: Contact investigations for antibiotic-resistant bacteria: a mixed-methods study of patients’ comprehension of and compliance with self-sampling requests post-discharge
Source: Antimicrob Resist Infect Control. 2023 Aug 10;12:77. doi: 10.1186/s13756-023-01277-1 (PMC10413776; doi:10.1186/s13756-023-01277-1)
Supplement: Supplementary file 2 — Additional file 2. Additional Tables. Basic characteristics of questionnaire respondents by self-reported compliance. Patients’ comprehension of the self-sampling request letter, asked by four different questions in the questionnaire, by CEFR-level of the received letter and educational level. [file 13756_2023_1277_MOESM2_ESM.docx]

**Additional file 2 Additional Tables.**

**Additional Table 1** Basic characteristics of questionnaire respondents by self-reported compliance (n=221).

|  | Respondents declaring compliance with self-sampling request (n=206)^b^ | | Respondents declaring non-compliance with self-sampling request (n=15) | |
| --- | --- | --- | --- | --- |
| **Respondents' characteristics** | **N** | **%** | **N** | **%** |
| **Median age (range)** | 68 (20-90) |  | 67 (39-83) |  |
| **Gender** |  |  |  |  |
| Male | 104 | 50.5 | 4 | 26.7 |
| Female | 101 | 49.0 | 11 | 73.3 |
| Other | 1 | 0.5 | 0 | 0.0 |
| **Living conditions** |  |  |  |  |
| Independently | 197 | 97.0 | 14 | 93.3 |
| In a healthcare facility | 6 | 3.0 | 1 | 6.7 |
| **Children <18 years living in household** |  |  |  |  |
| Yes | 19 | 9.7 | 2 | 13.3 |
| No | 176 | 90.3 | 13 | 86.7 |
| **Highest educational level** |  |  |  |  |
| No education | 4 | 2.0 | 1 | 6.7 |
| Primary school | 14 | 6.9 | 1 | 6.7 |
| High school | 85 | 41.9 | 9 | 60.0 |
| University of Applied Sciences | 85 | 41.9 | 4 | 26.7 |
| University | 15 | 7.4 | 0 | 0.0 |
| **(Previous) Healthcare worker**^a,b^ |  |  |  |  |
| Yes | 26 | 17.1 | 3 | 33.3 |
| No | 126 | 82.9 | 6 | 66.7 |

^a^ Due to the questionnaire layout, this question often went unnoticed.

^b^ Percentages are calculated based on the number of respondents per question.

**Additional Table 2** Patients’ comprehension of the contact investigation by CEFR-level and educational level.

|  |  | CEFR-level | No education  n (%) | Primary school n (%) | High school n (%) | University of Applied Sciences  n (%) | University  n (%) |
| --- | --- | --- | --- | --- | --- | --- | --- |
| Total number of patients |  |  | 6 | 13 | 82 | 78 | 16 |
| Was the screening for the detection of the bacterium clear at the time? | Yes | <B2 | 0 (0.0) | 7 (53.8) | 34 (41.5) | 25 (32.1) | 8 (50.0) |
|  |  | B2 | 5 (83.3) | 4 (30.8) | 38 (46.3) | 46 (59.0) | 8 (50.0) |
|  | No | <B2 | 0 (0.0) | 0 (0.0) | 2 (2.4) | 0 (0.0) | 0 (0.0) |
|  |  | B2 | 0 (0.0) | 1 (7.7) | 1 (1.2) | 3 (3.8) | 0 (0.0) |
|  | A little | <B2 | 1 (16.7) | 0 (0.0) | 2 (2.4) | 1 (1.3) | 0 (0.0) |
|  |  | B2 | 0 (0.0) | 1 (7.7) | 5 (6.1) | 3 (3.8) | 0 (0.0) |

*CEFR* Common European Framework of Reference language level.

**Additional Table 3** Patients’ comprehension of the information about the bacterium by CEFR-level and educational level.

|  |  | CEFR-level | No education n (%) | Primary school n (%) | High school n (%) | University of Applied Sciences n (%) | University n (%) |
| --- | --- | --- | --- | --- | --- | --- | --- |
| Total number of patients |  |  | 6 | 13 | 82 | 79 | 16 |
| Did you find the information about the bacterium clear at the time? | Yes | <B2 | 0 (0.0) | 6 (46.2) | 31 (37.8) | 22 (27.8) | 7 (43.8) |
|  |  | B2 | 4 (66.7) | 4 (30.8) | 33 (40.2) | 43 (54.4) | 6 (37.5) |
|  | No | <B2 | 0 (0.0) | 0 (0.0) | 3 (3.7) | 0 (0.0) | 0 (0.0) |
|  |  | B2 | 0 (0.0) | 1 (7.7) | 3 (3.7) | 5 (6.3) | 0 (0.0) |
|  | A little | <B2 | 1 (16.7) | 1 (7.7) | 4 (4.9) | 5 (6.3) | 1 (6.3) |
|  |  | B2 | 1 (16.7) | 1 (7.7) | 8 (9.8) | 4 (5.1) | 2 (12.5) |

*CEFR* Common European Framework of Reference language level.

**Additional Table 4** Patients’ comprehension of the self-sampling instructions by CEFR-level and educational level.

|  |  | CEFR-level | No education n (%) | Primary school n (%) | High school n (%) | University of Applied Sciences n(%) | University n (%) |
| --- | --- | --- | --- | --- | --- | --- | --- |
| Total number of patients |  |  | 6 | 13 | 81 | 79 | 16 |
| Did you find the information about taking the swabs clear at the time? | Yes | <B2 | 0 (0.0) | 7 (53.8) | 34 (42.0) | 25 (31.6) | 7 (43.8) |
|  |  | B2 | 5 (83.3) | 4 (30.8) | 39 (48.1) | 48 (60.8) | 8 (50.0) |
|  | No | <B2 | 0 (0.0) | 0 (0.0) | 2 (2.5) | 0 (0.0) | 0 (0.0) |
|  |  | B2 | 0 (0.0) | 1 (7.7) | 2 (2.5) | 2 (2.5) | 0 (0.0) |
|  | A little | <B2 | 1 (16.7) | 0 (0.0) | 1 (1.2) | 2 (2.5) | 1 (6.3) |
|  |  | B2 | 0 (0.0) | 1 (7.7) | 3 (3.7) | 2 (2.5) | 0 (0.0) |

*CEFR* Common European Framework of Reference language level.

**Additional Table 5** Patients’ comprehension of receiving results by CEFR-level and educational level.

|  |  | CEFR-level | No education n (%) | Primary school n (%) | High school n (%) | University of Applied Sciences n (%) | University n (%) |
| --- | --- | --- | --- | --- | --- | --- | --- |
| Total number of patients |  |  | 5 | 13 | 81 | 79 | 16 |
| Was it clear how you would get the results? | Yes | <B2 | 0 (0.0) | 6 (46.2) | 35 (43.2) | 23 (29.1) | 8 (50.0) |
|  |  | B2 | 3 (60.0) | 4 (30.8) | 31 (38.3) | 40 (50.6) | 8 (50.0) |
|  | No | <B2 | 0 (0.0) | 0 (0.0) | 2 (2.5) | 2 (2.5) | 0 (0.0) |
|  |  | B2 | 0 (0.0) | 1 (7.7) | 6 (7.4) | 6 (7.6) | 0 (0.0) |
|  | A little | <B2 | 0 (0.0) | 1 (7.7) | 1 (1.2) | 2 (2.5) | 0 (0.0) |
|  |  | B2 | 2 (40.0) | 1 (7.7) | 6 (7.4) | 6 (7.6) | 0 (0.0) |

*CEFR* Common European Framework of Reference language level.
